# Supplementary material for: The unmet mental health needs of adolescents with HIV in eastern Tanzania: Experiences of healthcare providers, adolescents, and caregivers
Source: PLoS One. 2024 Aug 28;19(8):e0307143. doi: 10.1371/journal.pone.0307143 (PMC11356450; doi:10.1371/journal.pone.0307143)
Supplement: S1 File — (DOCX) [file pone.0307143.s001.docx]

# S1. In-depth interview guide: Healthcare providers

1. How are you today?

Let’s begin by talking about your experience working with adolescents living with HIV, especially on psychological and mental health challenges.

1. Please tell me what you understand about psychological and mental health problems in adolescents living with HIV,

*(you may probe about depression, its causes, and its treatment).*

1. What about your perception of the effect of psychological and mental health problems like depression on the lives of adolescents living with HIV

*(Probe for effects on specific areas (e.g., home, school, HIV treatment, and overall quality of life)*

1. How do adolescents living with HIV Present when they have mental health problems?
2. What are the challenges you face as a provider to provide the care they need for mental health problems and related problems?

*(Probes: Are there any challenges in assessment, diagnosis, treatment, and care pathways eg. Referral to mental health expert?)*

1. What do you think should be the ideal treatment for these adolescents?

***You may query their knowledge of psychotherapy like CBT and the role of*** Psychologists & Psychiatrists in treatment.

**Transition Script:** *We are interested in how best to integrate brief psychological intervention in adolescent HIV care in other parts of Tanzania.*

1. What are the barriers to providing therapy?
2. Do you think therapy needs adapting? For example, ask about their opinion on the effect of culture and other factors on therapy for adolescents in Tanzania.

###

# **S2: In-depth Interview Guide: Caregivers**

1. How are you today?

Let’s begin by talking about your experience raising/living with an adolescent with HIV, especially on psychological and mental health challenges.

1. Please tell me what you understand about psychological and mental health problems in adolescents living with HIV, its causes, and its treatment.
2. How do adolescents with HIV Present when they have mental health problems

*(you may probe about depression and its symptoms)*

1. What about your perception of the effect of psychological/mental health problems like depression on the lives of adolescents with HIV?

*(Probe for effects on specific areas (e.g., home, school, HIV treatment, and overall quality of life)*

What are the challenges you face as a caregiver to deal with depression and related problems (Probe for challenges like adherence to ART).

1. Are you aware of any mental health services provided?
   1. Where? how did you find out?
   2. How easy is it for you to ask for it?
2. Have you ever attempted to seek help for adolescent mental health challenges?
   1. What happened? What went wrong? What went right?
   2. What are your thoughts about how friendly these services are to adolescents living with HIV?
3. Are there any challenges in care pathways?

(Probe: availability of mental health services, Referral to mental health expert)

1. What do you think should be the ideal treatment for these adolescents?

***You may query their knowledge of psychotherapy/CBT/***Psychologists & Psychiatrists.

**Transition Script:** *We are interested in how best to integrate brief psychological intervention in adolescent HIV care in other parts of Tanzania.*

1. What are your expectations from treatment or your experience with the implementation of treatment?
2. Do you have any experience with traditional methods of help with this condition?

# **S3: In-Depth Interview Guide – Adolescent**

**Thank you for agreeing to participate and completing the consenting process. I will now ask you more detailed questions about yourself, the treatment you have been receiving, and the challenges, if any.**

Can you tell me your experiences of the current health care that you have been receiving in this center?

*Remember to ask probing questions related to*

*(mental health/psychological services for psychological problems, emotional challenges, etc)*

1. Please tell me what you understand about psychological and mental health problems in adolescents living with HIV,

*(you may probe about depression, its causes, and its treatment).*

1. How do adolescents with HIV Present when they have mental health problems

*(you may probe about depression and its symptoms)*

1. What about your perception of the effect of psychological and mental health problems like depression on the lives of adolescents living with HIV

*(Probe for effects on specific areas (e.g., home, school, HIV treatment, and overall quality of life)*

1. Are you aware of any mental health services provided?
   1. Where? how did you find out?
   2. How easy is it for you to ask for it?
2. Have you ever attempted to seek help?
   1. What happened? What went wrong? What went right?
   2. What are your thoughts about how friendly these services are to adolescents living with HIV?
3. If you decide to seek help, what can be easy for you? difficult for you?

*Probe for barriers (e.g., time, shortage of providers, etc. logistical (e.g., distance, cost, hours of operation, etc; and informational (e.g., lack of awareness of services, lack of knowledge regarding the importance of services, etc*

**Transition Script:** *We are interested in how best to integrate brief psychological intervention in adolescent HIV care in other parts of Tanzania.*

1. What are your expectations from treatment or your experience with the implementation of treatment here at the HIV care and treatment center?
2. Do you have any experience of traditional methods that has helped you or someone you know or have been used to help of these mental health conditions?

*(Probe alternative remedies, religious rituals etc)*
