# Supplementary material for: The unmet mental health needs of adolescents with HIV in eastern Tanzania: Experiences of healthcare providers, adolescents, and caregivers
Source: PLoS One. 2024 Aug 28;19(8):e0307143. doi: 10.1371/journal.pone.0307143 (PMC11356450; doi:10.1371/journal.pone.0307143)
Supplement: Attachment — Submitted filename: Response to Reviewers.doc [file pone.0307143.s003.doc]

**SCHOOL OF MEDICINE**

***DEPARTMENT OF PSYCHIATRY AND MENTAL HEALTH***

P.O. Box 65001

DAR ES SALAAM

TANZANIA

Web: http://www.muhas.ac.tz

Mob: +255-717-547-606

Telefax: +255-22-2150465

E-Mail: tasiana2009@live.com

**MMUHIMBILI UNIVERSITY OF HEALTH AND ALLIED SCIENCES**

*MU/SM/PSY/9688/ 03.1/2023*

30^th^ ^TH^ July 2023

Editor-in-Chief

PLOS ONE

Dear Sir/Madam

**RE:	MANUSCRIPT RESUBMISSION AFTER ADDRESSING REVIEWERS’ COMMENTS**

We resubmit our manuscript entitled *“****The unmet mental health needs of adolescents living with HIV in Eastern Tanzania: Experiences of healthcare providers, adolescents, and Caregivers”*** by Njau et al. for consideration for publication as an Original Article in PLOS ONE after addressing comments from the editor and two reviewers as detailed below. Together with this letter, we are attaching two versions of manuscripts as required; one with corrections highlighted and the second as a clean version. However, we apologize that the draft with track changes has not comprehensively included all changes made, as the first reviewer forgot to enable the track change functionality. We have, however, highlighted most changes that were made in yellow. We appreciate the time and expertise of the Editor and reviewers, that has to this point greatly improved the quality of our manuscript.

**From the editor**

| **s/n** | **Editors’ comments** | **Authors response** | **Changes inserted in text on pg.no** |
| --- | --- | --- | --- |
| 1. | Include any set criteria to include or exclude interviewees. | Thank you for this observation. We have revised the manuscript and included criteria to include and exclude interviewees. | 5-6 |
| 2. | Clearly put your objectives | Thank you, we have improved the revised manuscript and put the objectives clear. | 2 and 4 |
| 3. | Specify which model of phenomenological qualitative study approach was employed | Thank you for this comment.  This study employed the **descriptive phenomenological qualitativ**e study whereby researchers wanted to understand the lived experiences of adolescents living with HIV in terms of psychological /mental health problems and care needs. | 2 |
| 4. | Explain how you managed bias (did you use bracketing?) | We thank you for the comment and question. To reduce respondents bias, we applied various interviewing techniques, and comparing opinions with those of other respondents and holding a meeting of HCPs to check the veracity of some of the opinions. Also, some participants were guarded during interviews, especially when discussing negative experiences with service delivery, out of fear of retaliation from the services.  We minimized this by ensuring confidentiality throughout the interview and using interviewers not involved in delivering their HIV care services. We acknowledge that since the interviews were conducted by a psychologist and social workers with mental health knowledge may introduce some bias. We used bracketing (probing the same questions differently and being naive on the topic) during data collection, multiple coders to interpret data, and triangulated data sources to minimized researchers’ bias. | 6 |
| 5. | Since your research is qualitative, try replacing terms such as "high level of unmet..." with equivalent qualitative vocabulary. | Thank you for this observation, we have revised and removed the term high levels | 2 |

**From Reviewer 1**

| **S/n** | **Reviewers’ comments** | **Authors response** | **Changes inserted on pg.no** |
| --- | --- | --- | --- |
| **--** | The authors are to be congratulated for considering this important subject. Unmet mental health needs are a challenging problem not only in people living with HIV but also in the general population, especially in low-income countries. Please address the following issues regarding your manuscript. | Thank you very much for the time used to review this manuscript and for providing valuable comments | **N/A** |
| 1. | The background of your manuscript needs rewriting. Because adolescents with HIV are not only at risk for depression but are also vulnerable to psychosis, mood spectrum disorder, etc. Make it more inclusive. | Thank you for this comment, we acknowledge that other common mental health problems were important. However, the study was part of a larger study, and this sub-study was purposely to inform the development of a psychological intervention for depression. We believe addressing depression which has a direct impact on ART adherence, will be an important entry point to explore other mental health challenges in the future.  We have, however, in addressing this comment, briefly added in the other mental health problems in the background. | 3-4 |
| 2. | You use a purposive sampling approach to recruit the participants. Which type of purposive sampling did you use? Specify it. | Thank you for this comment.  We used the Maximum variation sampling technique to arrive at the selected participants. We have reviewed the sampling procedure and included the type of purposive sampling and its justification. | Pg 4-5 |
| 3. | Since you have used human participants, instead of saying NA, you have to state the ethics statement from the online submission of your manuscript. | Thank you. We have revised the ethics statement during the online submission process. | - |
| 4. | In your data availability statement, you have stated that all relevant data are within the manuscript and its supporting information, but your manuscript lacks supporting information. Please include the supporting information in your manuscript. | Thank you for this observation; we have now attached all supporting information including in-depth interview guides for adolescents, health care providers and care givers. We have also included the Consolidated criteria for reporting qualitative studies (COREQ): 32-item checklist that guided the reporting of this study findings. | Supporting files |
| 5. | Please include a caption for Table 1. | Thank you for this observation. We have included caption for Table 1 | Pg 10 |
| 6. | I note you have included a table to which you do not refer in the text of your manuscript. Please ensure that you refer to table 1 in your text. | Thank you. We have removed the social demographic distribution table because its description is in the text. | Pg 9 |
| 7. | Other problems are minor. The paper could be improved grammatically by using a native English speaker and lack of publication information for reference number 32. | Thank you for highlighting this. We have taken time to read and peer-edited the manuscript to address the grammatical errors. We have also crosschecked and updated the references | 1-33 |

**From reviewer 2**

| **s/n** | **Reviewers’ comments** | **Authors response** | **Changes inserted in text on pg.no** |
| --- | --- | --- | --- |
| 1. | Why ethical review board of AAU needed? since the study was in Tanzania? justify | We thank you for the questions.  The study is part of a Ph.D programme and the first author is registered at Addis Ababa University and hence required an Ethical clearance from Ethiopia Review Board. | - |
| 2. | It is interesting finding but would have sounded better if you had mixed with quantitative study design | Thank you for the comment. This work was part of a larger study about psychological intervention for depression. The current manuscript aimed to inform the intervention development theory of change meetings. We have however, in addressing this comment acknowledged this in the limitation section. | Pg 18 |
| 3. | Why are only depression symptoms experienced? why not psychotic symptoms? or anxiety, how about substance use disorder and related mental illness? | We thank you for the questions.  We acknowledge that other common mental health problems were important. However, the study was part of a larger study, and this sub-study was purposely to inform the development of a psychological intervention for depression. We believe addressing depression which has a direct impact on ART adherence will be an important entry point to explore other mental health challenges in the future. We have however in addressing this comment included a general statement about other mental health conditions in the introduction. | 2 and 3 |
| 4. | Sample size did not look based on saturation, if so, how did it happen to be equal among each participant? by change? justify | We thank you for this observation and your questions. We have revised the manuscript and added an explanation to this. The proposed sample size for which Ethical clearance for this study was granted was 45 interviews (15 for each subgroup) however, saturation of data was achieved at 12 interviews for adolescents, 13 interviews for healthcare providers and 9 interviews for caregivers. Since the actual number of interviews at which saturation occurred was close to what was approved by Ethics board, we continued to 15 per category to check if new information will arise. | Pg 7 |
| 5. | I believe that not having quantitative should be limitation of your study, you may do this as limitation | Thank you for this comment. We agree that these findings could have been strengthened by quantitative data. However, this manuscript highlights results of a larger study aiming at informing the second phase on theory of change workshops for developing a psychological intervention (published work). Quantitative data was collected in subsequent phase three and will be reported in a pilot RCT study that is currently on manuscript development stage. However, in addressing this comment, we have acknowledged the lack of quantitative data in the limitation. | Pg 18 |
| 6. | I recommend to attach your questionnaires as annex, others may refer it when needed. | Thank you for this comment; we have attached in-depth interview guides for adolescents, health care providers and care givers. | Supplementary material |

Sincerely,

Tasiana Njau

Ph.D. Candidate, Addis Ababa University

Assistant Lecturer, Department of Psychiatry & Mental Health

Muhimbili University of Health and Allied Sciences
